# Design and implementation of an intelligent system for smart garden watering using fuzzy logic

**DOI:** 10.3389/frai.2026.1789499

**Published:** 2026-06-26

**Authors:** Hind Mestouri, Kamal Baraka, Jamal Ezzahar, Mohamed Madiafi

**Affiliations:** 1 Cadi Ayyad University (UCA), National Schools of Applied Sciences (ENSA) in Safi, Laboratory of Mathematical Computer and Communication System, Safi, Morocco; 2 Laboratory of Fluids and Energy Mechanics (LMFE), National Schools of Applied Sciences (ENSA) in Safi/Center for Remote Sensing Applications, Université Mohammed VI Polytechnique (UM6P), Ben Guerir, Morocco

**Keywords:** Arduino microcontroller, cloud, FUZZY LOGIC, internet of things (IoT), mobile application, smart garden

## Abstract

This paper presents an intelligent automated watering system for residential gardens, based on fuzzy logic, an Arduino microcontroller, and Internet of Things (IoT) technology with cloud integration. The system relies on cost-effective and readily available hardware components, including ambient temperature and soil moisture sensors, to continuously monitor garden in real time. A functional prototype was developed to evaluate the practical performance of the proposed solution. Sensor data are processed using fuzzy logic, allowing the system to make adaptive decisions for garden maintenance. According to the input variables, the system automatically activates appropriate actuators, such as a heating bulb, a water pump, or a fan, to optimize garden watering. The system includes an Android mobile application that enables direct Wi-Fi connectivity for real-time monitoring and control. Additionally, a cloud-based platform is integrated to ensure secure data storage, visualization, and remote access. This enables users to monitor irrigation status, and receive alerts even when away from home. Experimental results show that the proposed system reduces irrigation water consumption compared with conventional threshold-based control, while maintaining soil moisture within the optimal range. These results confirm the effectiveness of the proposed solution, highlighting the successful use of the fuzzy logic-based control system in combination with the mobile application. This integration enables adaptive decision-making and efficient monitoring, resulting in a practical and cost-efficient solution for home garden management.

## 1 Introduction

Technology has become fundamental to modern life, driving transformative change across multiple sectors, particularly agriculture. As global demand for efficient resource management increases, researchers are actively developing innovative solutions to improve water-use efficiency. In this context, intelligent automation systems have emerged as essential tools for effective water resource management (Jha et al., 2019; Ray, 2017). Urban residents increasingly face challenges in maintaining home gardens, especially when travel or busy schedules limit consistent care. This growing disconnection from nature has intensified interest in automated and intelligent gardening solutions that are both affordable and remotely controllable via smartphones.

The Internet of Things (IoT) has emerged as a key enabling technology in this domain, allowing real-time monitoring, control, and interaction with physical gardening systems through seamless digital connectivity (Sharma et al., 2019). IoT frameworks facilitate data-driven interactions between users and their environments. Modern smart garden systems rely on integrated sensor networks that combine hardware, software, and standardized communication protocols to enable automatic operation (Misra et al., 2016). However, as highlighted by Chen (2012), widespread IoT adoption continues to face significant challenges related to scalability, system reliability, and interoperability, despite its strong potential for automation and environmental management. A major challenge for home gardeners is ensuring appropriate irrigation and plant care, as inconsistent maintenance often results in dehydration or overwatering. IoT-based smart garden systems address this issue by dynamically regulating environmental conditions to support plant health and optimize growth (Deore et al., 2018; Choudhari et al., 2023; Ali et al., 2020). These systems monitor key parameters such as soil moisture, ambient temperature, water reservoir levels, and light exposure, which naturally vary over time and directly influence plant growth and water requirements (Looi and Rahmat, 2022; Mukhlisin et al., 2021). Although commercial smart garden solutions are available, their high cost and limited functionality restrict accessibility for many users.

To overcome these limitations, this study proposes a low-cost, multifunctional Smart watering system based on Arduino microcontroller (Angal and Mali, 2016). The system automatically determines optimal irrigation schedules by analyzing real-time soil moisture data, while simultaneously monitoring ambient temperature to regulate microclimate conditions. Real-time data are transmitted to both a mobile application and a cloud platform, reducing the need for manual intervention and enabling long-term data logging, historical trend visualization, and future predictive analysis. The system employs fuzzy logic to process inputs from soil moisture and temperature sensors, enabling dynamic adjustment of water supply via an adaptive decision-making mechanism (Berenji, 1992; Kaur and Singh, 2017; Abdullah et al., 2020). An accompanying Android application enables remote monitoring via Wi-Fi, providing live sensor readings and notifications to keep users informed about the garden's status (Ali et al., 2020). Cloud integration further enhances the system by supporting secure data storage, multi-user access, and potential future integration with weather forecasting services or machine learning models (Pargo et al., 2025; Nabaei et al., 2025).

This research presents an intelligent, automatic irrigation system specifically designed for home gardens. By integrating fuzzy logic-based adaptive control with mobile and cloud-enabled remote monitoring, the proposed system ensures reliable and efficient plant maintenance, even during extended periods of user absence. The solution emphasizes affordability, scalability, and sustainability, thereby contributing to accessible smart garden technologies and advancing resource-efficient home garden management.

## 2 Literature review

A comprehensive analysis of existing literature reveals significant advancements in irrigation automation technologies for residential gardens. Current research emphasizes the integration of multisensor systems with advanced decision-making algorithms to maximize the capabilities of the IoT. Notably, recent smart garden architectures employ standardized communication protocols to facilitate remote monitoring through web-based platforms and mobile applications (Ali et al., 2020). Such systems provide adaptive, data-driven solutions that serve the needs of both research communities and practical end-users. Several studies have explored IoT-enabled automated irrigation systems built on sensor networks and microcontroller platforms. These solutions often integrate microcontrollers such as Arduino or Raspberry Pi with multiple sensors to enable automatic plant monitoring and maintenance. For example, Angal and Mali (2016) describes a hydraulic irrigation system incorporating Arduino, Raspberry Pi, and IoT modules for soil moisture and water level monitoring, featuring smartphone-accessible data visualization. Ramli et al. (2023) presents an indoor gardening system employing Raspberry Pi with multimodal sensors, including infrared, gas, and ultrasonic sensors. Thamaraimanalan et al. (2018) extends this approach to larger gardens by incorporating environmental sensors, such as humidity and temperature, for precision irrigation control. Meanwhile, an intelligent plant watering system built on IoT and artificial intelligence (AI) was proposed to deliver regular updates and reports based on weather conditions (Penzenstadler et al., 2018).

In parallel, recent developments in smart urban gardening systems have introduced diverse technological strategies for irrigation optimization. Robotic systems such as PlantPal employ precision sensors for fully remote garden management (Zeqiri et al., 2025), while other implementations utilize fuzzy logic controllers integrated with soil moisture and precipitation sensors to dynamically optimize watering schedules (Ambarwari et al., 2024; Arginanta and Ashari, 2024). The effectiveness of fuzzy logic in enhancing irrigation efficiency under variable environmental conditions has been validated in recent studies (Abdullah et al., 2020; Kaur and Singh, 2017). Some irrigation monitoring systems primarily rely on local storage of sensor data, which can limit data accessibility and long-term analysis capabilities (Singh et al., 2018; Van et al., 2022; Ritesh and Jabeena, 2016). In contrast, several recent studies have adopted cloud-based platforms to support real-time monitoring, data visualization, and remote access to system information, thereby extending system functionality beyond local monitoring (Morchid et al., 2024; Ali et al., 2020; Tumpa et al., 2023). Complementing these systems, mobile applications now provide intuitive interfaces for real-time monitoring and simplified user interaction with automated gardening platforms (Rashid et al., 2025).

Recent research trends highlight the adoption of hybrid AI models and multimodal sensing to improve decision accuracy. Vision-based systems can assess plant health and predict water stress, while agent-based approaches enhance scalability and resource allocation (Nabaei et al., 2025; Pargo et al., 2025). Building on this foundation, several studies have explored related irrigation solutions. A GSM-enabled Arduino-based irrigation prototype that sends SMS notifications to users was introduced in Singh et al. (2018). Another work employed wireless sensor networks to create an IoT-enabled irrigation system that improved both water usage and energy efficiency (Van et al., 2022). To reduce wiring complexity and enhance scalability, ZigBee-based wireless communication between sensors and a central controller was adopted in Ritesh and Jabeena (2016). A fuzzy logic-based irrigation control system using membership functions for key environmental parameters was proposed in Kaur and Singh (2017), demonstrating improved performance over simple threshold-based approaches. Bluetooth-enabled systems, such as the one presented in Tasayco et al. (2025), provide an intuitive mobile interface for managing small urban gardens. Although limited in communication range, such systems remain practical for compact environments. A solar-powered irrigation system using low-power sensors and ESP8266 microcontrollers was introduced in Kumar et al. (2023), making it suitable for off-grid deployments. The integration of cloud computing to enhance irrigation control through real-time dashboards and alerts was demonstrated in Morchid et al. (2024).

Machine learning techniques, particularly convolutional neural networks (CNNs), have been applied to leaf image analysis for plant health assessment and automated irrigation decision-making (Boulent et al., 2019). Although computationally demanding, this approach highlights the potential of vision-based crop monitoring. Commercial solutions such as Xiaomi Mi Plant Flower Monitor and RainMachine also provide sensor-based systems with mobile integration, but these platforms are often proprietary and costly (Smart H. E., 2024). In contrast, open-source platforms based on Arduino and Raspberry Pi offer affordable and customizable alternatives for researchers and hobbyists. The importance of user-friendly interfaces has also been emphasized, with real-time visualization and intuitive mobile applications shown to significantly improve the adoption of Smart watering systems among non-technical users (Gutiérrez et al., 2013). In summary, although existing research presents a wide range of automated irrigation approaches, only a limited number of systems integrate a microcontroller-based architecture with fuzzy logic control and mobile application support for residential home gardens. To address this gap, this study proposes a low-cost, adaptive smart garden system built on an Arduino platform, where fuzzy logic serves as the core decision-making mechanism, complemented by comprehensive mobile application support and cloud integration for extended monitoring and data analysis.

## 3 Methodology

### 3.1 Architecture of the automatic smart garden watering system

**Figure 1:**
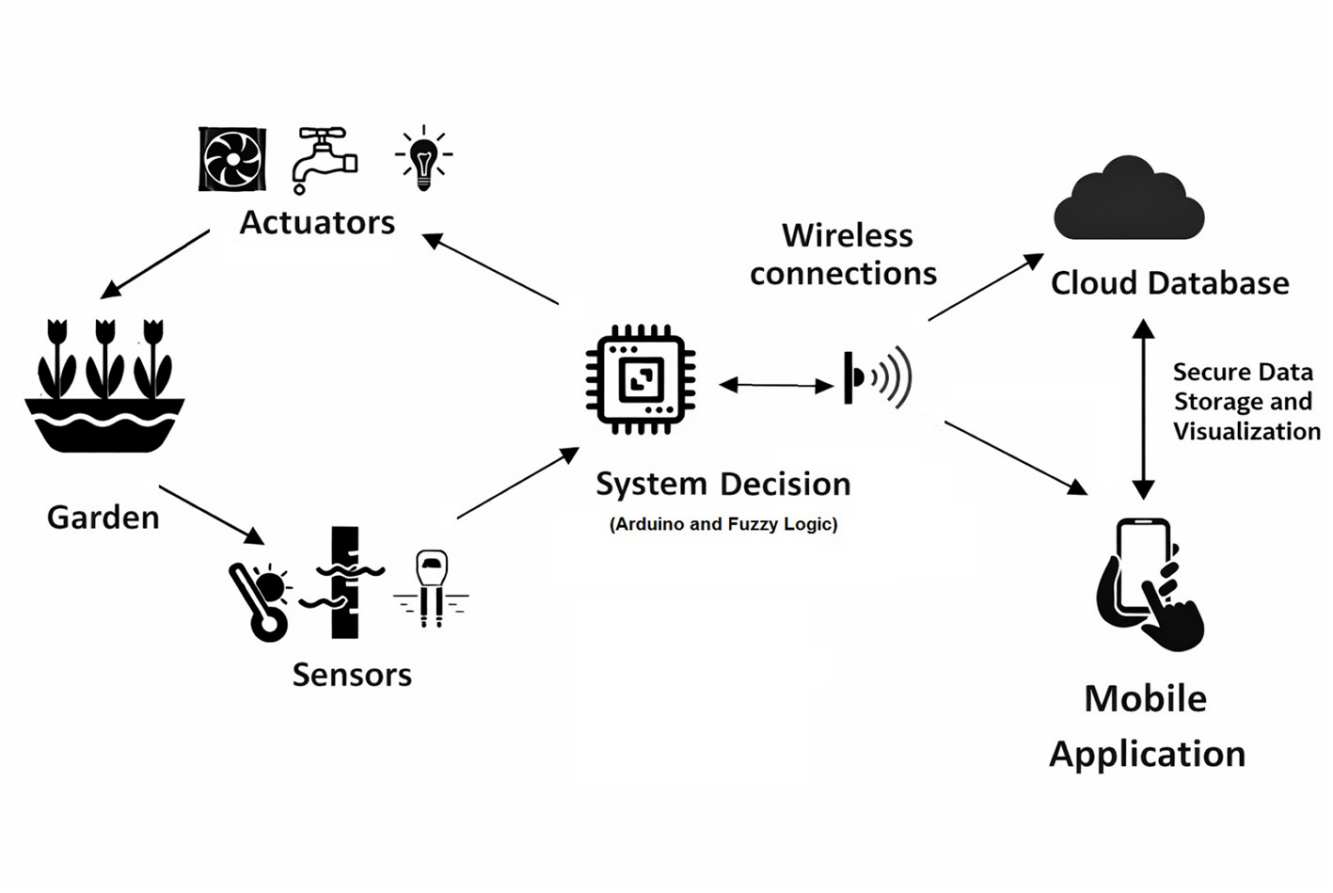
Block diagram of the automatic irrigation system, showing the interaction between sensors, decision-making unit (Arduino and fuzzy logic), actuators, wireless communication, cloud database, and mobile application for monitoring and control.

The overall architecture of the proposed automatic smart garden watering system is illustrated in Figure 1. Environmental sensors deployed in the garden continuously collect data related to soil moisture, ambient temperature, and water levels. These parameters constitute the core inputs of the fuzzy logic controller and are used for autonomous decision-making. The selection of these input variables was guided by their relevance to irrigation requirements and system reliability. Soil moisture directly reflects water availability in the root zone and is the primary indicator of plant water stress Foth (1990). Ambient temperature influences evapotranspiration and water demand, making it an important complementary factor in irrigation decisions Ray (2017). The water level in the reservoir is included as an operational parameter to ensure safe system operation and prevent pump dry-running Abdullah et al. (2020). These variables were selected as the most essential indicators of irrigation status while maintaining a simple and cost-effective system design. Additional environmental information, such as rainfall data, is not measured locally but retrieved from a nearby weather station and used exclusively for experimental analysis and validation purposes rather than real-time control. These sensor readings are transmitted to the decision-making unit, which consists of an Arduino microcontroller implementing a fuzzy logic-based control algorithm. Based on the processed data, the system generates appropriate control actions to activate the actuators, such as the water pump, fan, or lighting elements.

At the same time, the data and system status are sent via Wi-Fi to a mobile application for real-time monitoring and user interaction. In parallel, the Arduino controller, connected to a Wi-Fi module, transmits sensor data to the ThingSpeak cloud platform using the MQTT (Message Queuing Telemetry Transport) protocol, a lightweight communication protocol widely used in IoT applications. In this setup, the Wi-Fi module acts as an MQTT client, sending sensor readings to dedicated channels on ThingSpeak. The platform then stores and processes the data, providing real-time visualization as well as access to historical records through interactive dashboards. This cloud-based approach ensures efficient data transmission with low bandwidth usage and enables remote access to the system from anywhere, improving both scalability and usability.

**Figure 2:**
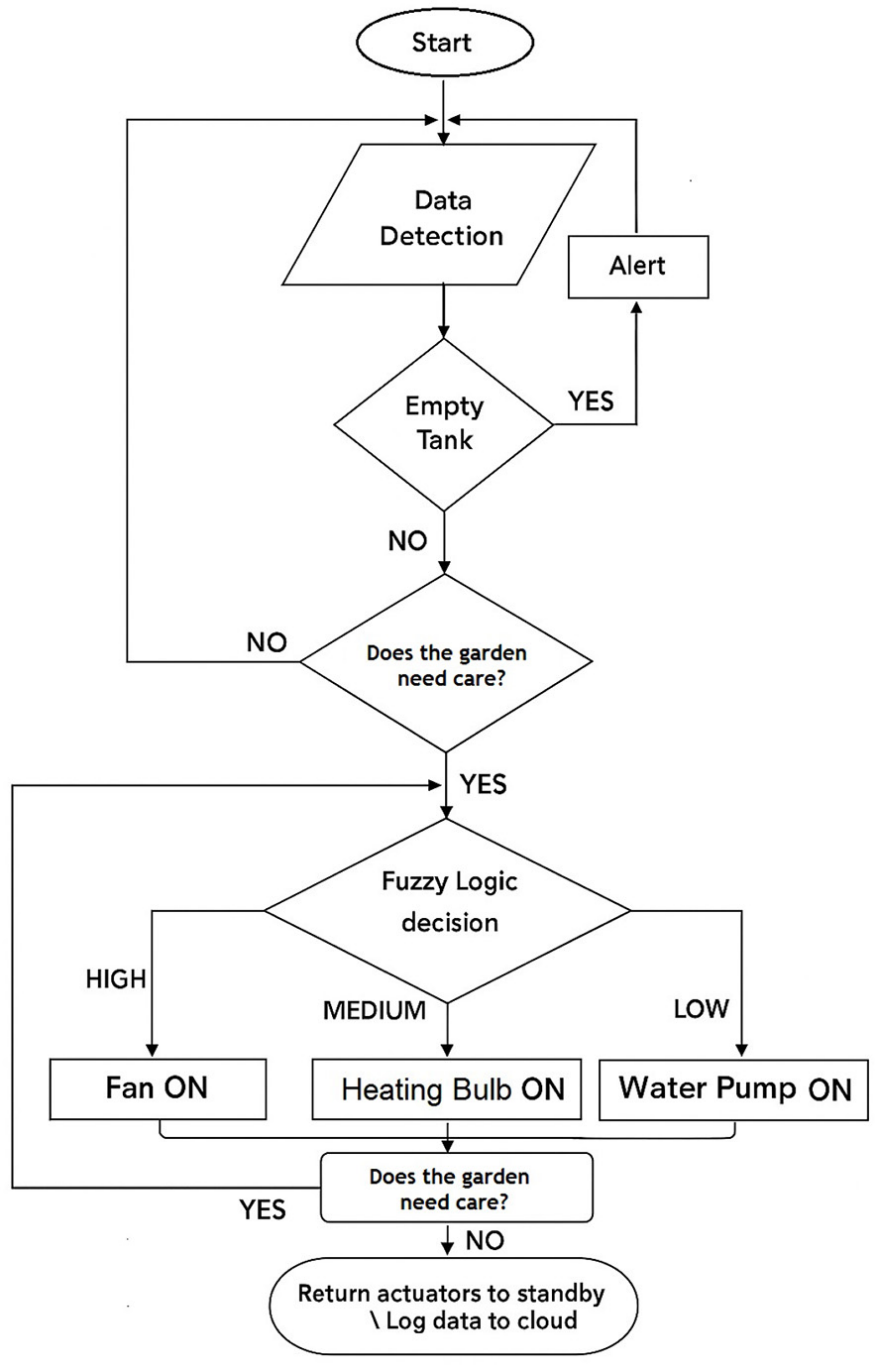
Workflow of the proposed system.

System operation begins with continuous monitoring of the water reservoir level using a dedicated sensor. If the available water level falls below a predefined threshold, the system suspends all irrigation activities and enters a waiting state until the reservoir is replenished. Once a sufficient water level is detected, the system evaluates soil moisture and ambient temperature conditions. If both parameters fall within acceptable ranges, the system remains in standby mode. When abnormal conditions are detected, the system transitions to the decision-making stage, where fuzzy logic is applied to interpret sensor inputs and determine appropriate actions. The complete operational workflow of the system is summarized in Figure 2, which illustrates the automated control sequence. Overall, the proposed system integrates hardware components with a communication framework and an intelligent control strategy. Its implementation consists of three main components: electrical hardware, fuzzy logic-based decision-making, and a mobile application interface. Once environmental conditions return to acceptable levels, the system automatically stops active control and resumes normal monitoring operations, ensuring efficient garden management.

### 3.2 Fuzzy logic and decision method

The proposed system employs a fuzzy logic-based control strategy to optimize home garden maintenance under varying environmental conditions. Based on real-time sensor measurements, the controller activates the appropriate actuators to regulate irrigation, heating, or ventilation according to plant needs. Sensor data are processed using a rule-based fuzzy inference mechanism that enables adaptive and context-aware decision-making. The fuzzy logic controller was designed using a limited number of input variables to maintain simplicity and interpretability in a low-cost embedded implementation. Soil moisture and ambient temperature were selected as the primary agronomic inputs since they are directly related to plant water demand. The water tank level was included as an operational constraint to prevent irrigation when water availability is insufficient and to protect the pumping system. This configuration enables efficient irrigation decision-making while minimizing hardware complexity and preserving system reliability.

**Figure 3:**
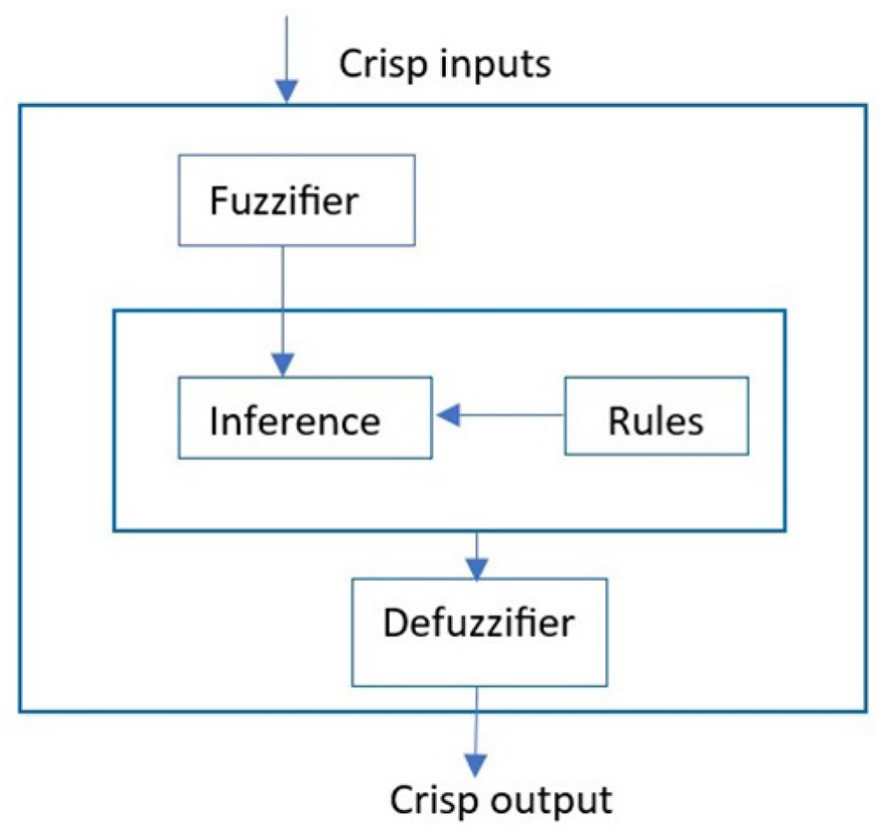
Architecture of the proposed fuzzy logic system.


The controller performance was evaluated through simulations using the MATLAB Fuzzy Logic Toolbox to test different rule structures and membership function parameters. The fuzzy inference system consists of three main stages: fuzzification, rule evaluation, and defuzzification. The control output is obtained using centroid defuzzification, selected for its balance between computational simplicity and accuracy. The overall architecture of the proposed fuzzy logic system is illustrated in Figure 3. In general, fuzzy logic is based on the concept of a fuzzy set (Berenji, 1992). A fuzzy set *S* is defined as a pair (*U, m*), where *U* represents the universe of discourse and *m*_*S*_ is the membership function, with *m*_*S*_:*U* → [0, 1]. In this framework, both the universe of discourse and the membership function play a critical role. As described in Sowah (2019) and expressed in Equation 1, a fuzzy set can be represented as a collection of ordered pairs, each consisting of an element *e* and its corresponding membership value:

$$\begin{array}{l} S=\left\{\left(e,m_{S}\left(e\right)\right)|e\in U\right\} \end{array}$$ (1)

The membership function *m*_*S*_(*e*) indicates the degree or probability that element *e* belongs to the set *S*. In the proposed system, three fuzzy subsets of *S*, namely *A*, *B*, and *C*, are defined. As shown in Equations 2, 3, *m*_*A*_(*x*), *m*_*B*_(*x*), and *m*_*C*_(*x*) represent the degrees to which an element *x* belongs to sets *A*, *B*, and *C*, respectively (Sarwar et al., 2018). In this context, the variables *A*, *B*, and *C* correspond to ambient temperature, soil moisture and water level. These equations define the fuzzy union and intersection of the two sets:


$$\begin{array}{l} A\cup B\cup C=\left\{\left(x,\max\left(\left(m_{A}\left(x\right)\right),m_{B}\left(x\right)\right),m_{C}\left(x\right)\right)|x\in S\right\} \end{array}$$ (2)

$$\begin{array}{l} A\cap B\cap C=\left\{\left(x,\max\left(\left(m_{A}\left(x\right)\right),m_{B}\left(x\right)\right),m_{C}\left(x\right)\right)|x\in S\right\} \end{array}$$ (3)

In the proposed approach, the rule strength is computed based on the fuzzy set operations defined in Equations 2, 3. The union operation in Equation 2 implements the fuzzy “*OR”* operator, which combines fuzzy inputs by selecting the maximum value of their membership degrees. In contrast, the intersection operation in Equation 3 applies the fuzzy “*AND”* operator by taking the minimum value. This operation determines the rule strength during the inference process. Fuzzy sets provide an effective framework for representing linguistic variables. Each element within the universe of discourse is assigned a membership value in the interval [0, 1]. In the proposed system, domain knowledge is encoded using fuzzy “*IF–THEN*” rules, which form the core of the decision-making mechanism. The inference engine subsequently applies a rule base composed of expert-defined fuzzy rules to generate fuzzy conclusions from the fuzzified inputs. Finally, the defuzzification stage converts the resulting fuzzy outputs into crisp control signals using suitable defuzzification techniques. Each component of the proposed fuzzy logic system is described in detail in the following subsections.

#### 3.2.1 Description of fuzzification

Fuzzification maps crisp sensor inputs into fuzzy sets using predefined membership functions. In this study, the input variables *rate of change of ambient temperature* and *soil moisture* are described using the linguistic terms *Low, Medium*, and *High*, while *water level* is represented by *Full* and *Empty*. Each linguistic label corresponds to a numerical range mapped to membership values in the interval [0, 1], allowing the system to handle uncertainty and gradual transitions between environmental conditions. The fuzzified representation is defined by Equation 4.

$$\begin{array}{l} Ã=\mu_{1}K\left(x_{1}\right)+\mu_{2}K\left(x_{2}\right)+\cdots +\mu_{n}K\left(x_{n}\right) \end{array}$$ (4)

The fuzzy set *K*(*x*_*i*_), referred to as the *fuzzification kernel*, represents the mapping of a crisp input value *x*_*i*_ to its fuzzy form. In this formulation, μ_*i*_ denotes a constant associated with the input value, and the crisp variable *x*_*i*_ is mapped to the fuzzy set *K*(*x*_*i*_). In the proposed system, the fuzzification process is governed by Equation 4, where the universe of discourse and the associated membership functions are systematically defined, enabling robust handling of uncertainty in the control process (Sarwar et al., 2018).

#### 3.2.2 Membership function design

**Figure 4:**
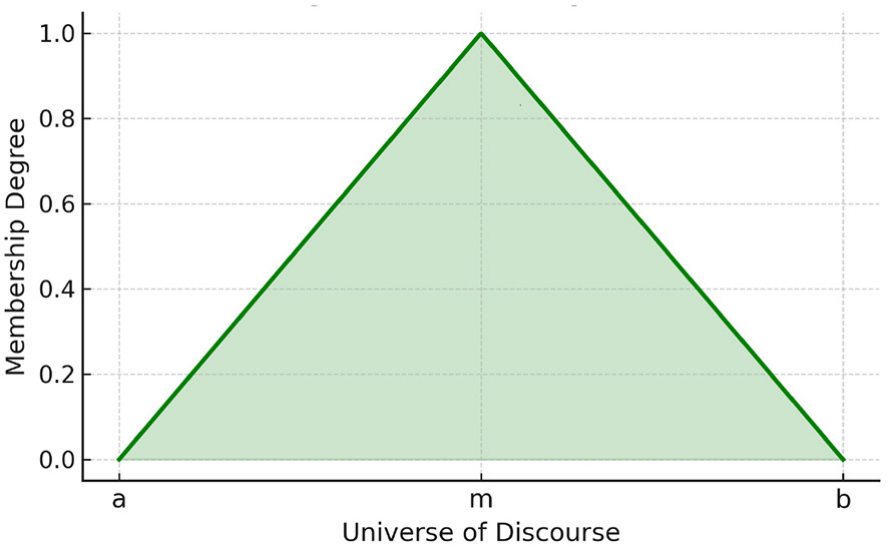
Triangular membership function.


The membership function of a fuzzy set *A* is defined as μ_*A*_:*X* → [0, 1], where *X* is the universe of discourse. In this study, triangular membership functions are adopted due to their simplicity and ease of implementation in the MATLAB Fuzzy Logic Toolbox, where they are implemented using the “*trimf* ” function. Each function is defined by three parameters (*a, m, b*), representing the lower bound, the peak, and the upper bound, with *a*<*m*<*b*, as shown in Figure 4 and expressed in Equation 5 (Foth, 1990).

$$\begin{array}{l} \mu_{A}\left(x\right)=\{\begin{array}{cc} 0, & x\leq a,\\ \frac{x-a}{m-a}, & a<x\leq m,\\ \frac{b-x}{b-m}, & m<x<b,\\ 0, & x\geq b. \end{array} \end{array}$$ (5)

As illustrated in Figure 4, the triangular membership function is defined over the input domain, with the membership degree ranging between 0 and 1. Equation 6 provides an equivalent formulation of Equation 5 using the *min* and *max* operators.


$$\begin{array}{l} \text{Triangle}\left(x;a,m,b\right)=\max\left(\min\left(\frac{x-a}{m-a},\frac{b-x}{b-m}\right),0\right) \end{array}$$ (6)

The parameters (*a, m, b*) define the triangular membership functions used for the three input variables (*ambient temperature variation, soil moisture variation*, and *water level*) as well as the output variable *garden needs care*. These functions were implemented in the MATLAB Fuzzy Logic Toolbox (Sarwar et al., 2018).

#### 3.2.3 Fuzzy inference system

Fuzzy inference combines membership functions, a rule base of *IF–THEN* statements, and logical operators (*AND, OR*) to generate control decisions. In this study, the Mamdani approach is adopted for its interpretability and suitability for rule-based systems (Almeida et al., 2023). The inference process evaluates the activated rules based on their firing strength, aggregates their outputs, and produces a unified fuzzy output, which is then converted into a crisp control signal through defuzzification.

$$\begin{array}{l} \text{IF}\left(x_{1}\text{is}A_{1}^{1}\;\text{AND}y_{1}\text{is}A_{2}^{1}\;\text{AND}z_{1}\text{is}A_{3}^{1}\right)\;\text{THEN}\left(w\text{is}B^{1}\right) \end{array}$$ (7)

$$\begin{array}{l} \text{IF}\left(x_{2}\text{is}A_{1}^{2}\;\text{AND}y_{2}\text{is}A_{2}^{2}\;\text{AND}z_{2}\text{is}A_{3}^{2}\right)\;\text{THEN}\left(w\text{is}B^{2}\right) \end{array}$$ (8)

**Figure 5:**
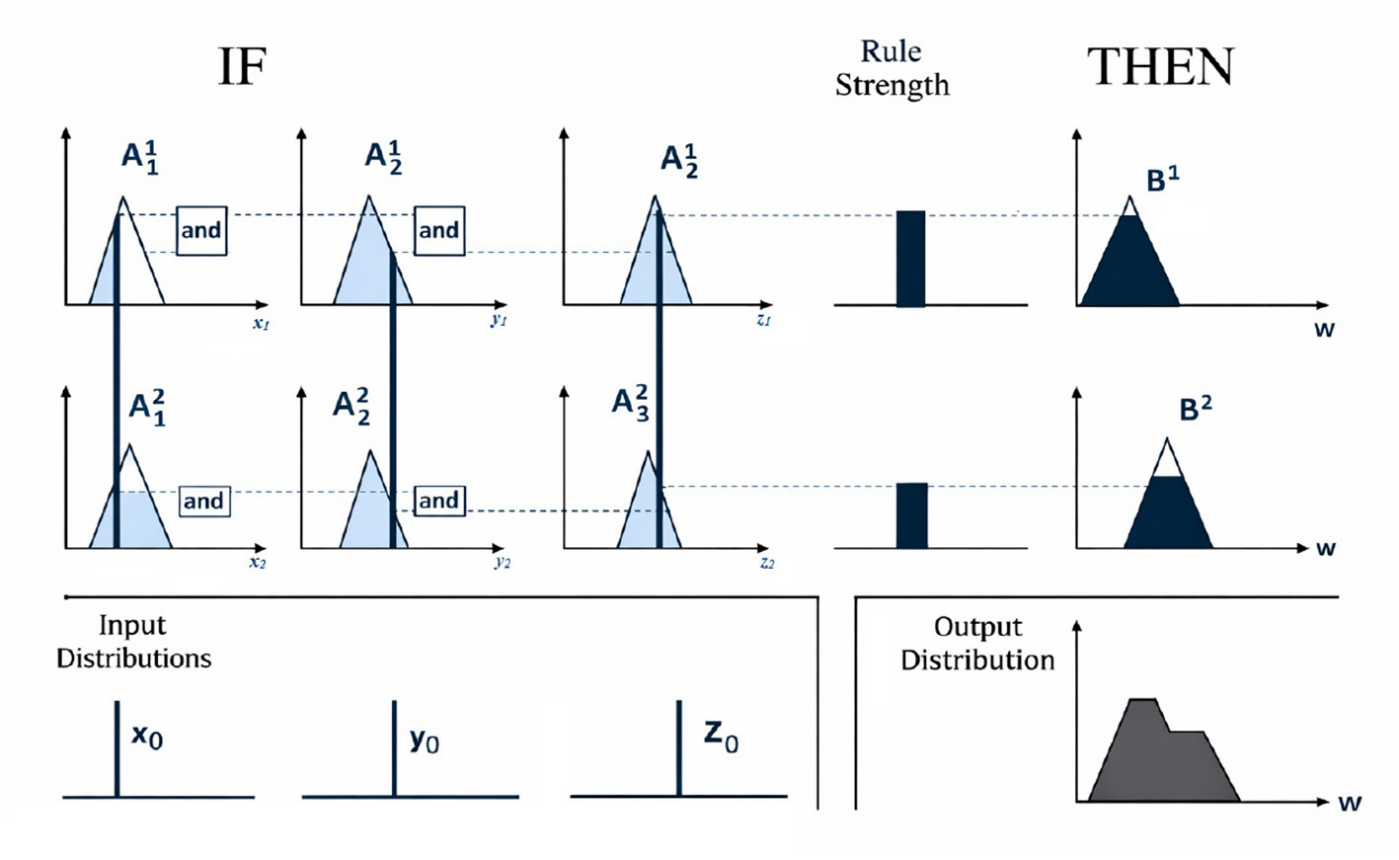
Mamdani inference system with three inputs, two rules, and crisp input values. Source: Symmetry (MDPI), Open Access. License: CC BY 4.0. (Sarwar et al., 2018).

In the proposed approach, Mamdani's fuzzy inference system is configured with three input variables and a set of two fuzzy rules. The fuzzified inputs, illustrated in Figure 5 and denoted as *x*_1_, *y*_1_, and *z*_1_, are defined according to Equations 7, 8. Each input is evaluated based on its membership function and the corresponding crisp sensor value. The fuzzy “*AND*” operator is employed to combine the fuzzified input sets when constructing the inference rules. As described in (Guillaume 2001), each fuzzy rule is associated with an output membership function, and its firing strength, representing the degree of truth of the rule, is determined by the current system state.

#### 3.2.4 Defuzzification

Defuzzification is the final stage of the fuzzy logic control system, where aggregated fuzzy outputs are converted into a single crisp control value (Sarwar et al., 2018). Among various methods, the centroid approach is adopted due to its accuracy and robustness. The process is implemented using the MATLAB Fuzzy Logic Toolbox, with its mathematical formulation provided in Equation 9.


$$\begin{array}{l} x^{*}=\frac{\int x\mu_{i}\left(x\right)dx}{\int \mu_{i}\left(x\right)dx} \end{array}$$ (9)

**Figure 6:**
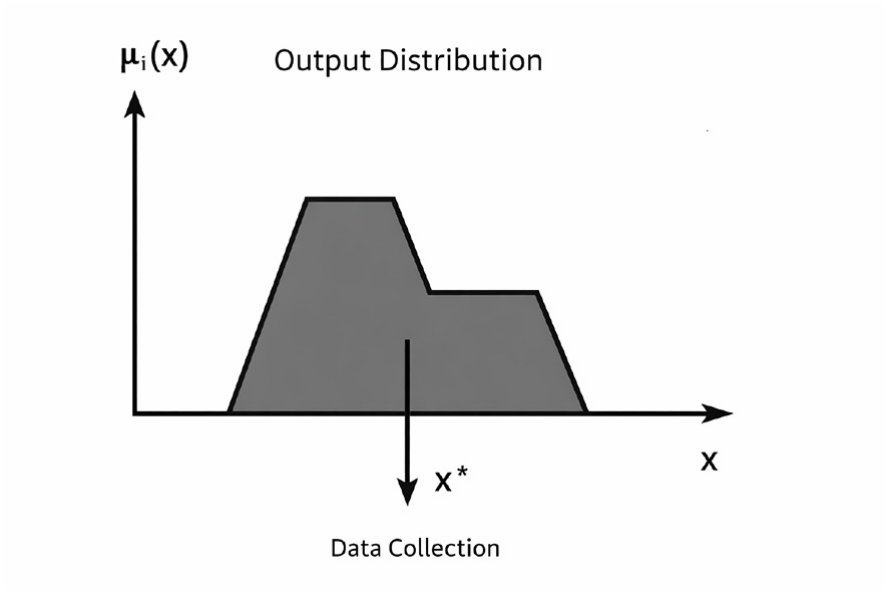
Resulting output computed using the centroid distribution approach. Source: Symmetry (MDPI), Open Access. License: CC BY 4.0. (Sarwar et al., 2018).

In Equation 9, x^*^ denotes the defuzzified output obtained from the aggregated membership function μ_*i*_(*x*) of the output variable. As illustrated in Figure 6, the horizontal axis represents the output domain, while the vertical axis corresponds to the membership grade. The final crisp control value x^*^ is computed as the centroid of the aggregated output, formed by scaling the membership functions according to the firing strength of the activated rules.

### 3.3 Implementation of automatic smart garden watering system

The intelligent watering system's hardware architecture comprises the following components:

• Arduino Uno

**Figure 7:**
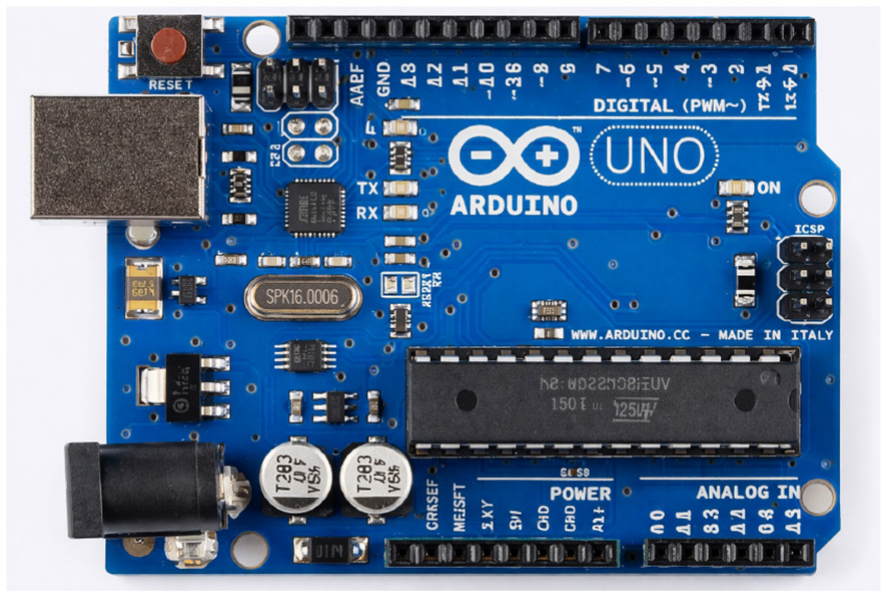
Arduino Uno microcontroller board used in the proposed system.

Among the various available Arduino boards, the Arduino Uno (Figure 7) was selected due to its wide adoption, reliability, and suitability for the proposed system (Arduino, 2025a). It is based on an Atmel AVR 8-bit microcontroller operating at 16 MHz and is supported by an open-source development environment, which facilitates efficient signal processing and control of connected sensors and actuators (Arduino, 2025b). Compared with alternative platforms such as Raspberry Pi or PIC-based controllers, the Arduino Uno offers strong compatibility with low-cost sensors, extensive community support, and high affordability, making it particularly well suited for prototyping and educational applications (Singh et al., 2018). The control firmware is implemented in C language and deployed using Arduino IDE v1.8.19, enabling reliable real-time execution on the ATmega328P microcontroller. During operation, sensor data are monitored via the serial interface and exported for MATLAB-based validation, ensuring seamless integration between data acquisition, fuzzy decision-making, and actuator control.

• Soil moisture sensor (YL-69)

Soil moisture is measured using the YL-69 sensor, chosen for its low cost, simple integration, and acceptable accuracy for garden-scale monitoring (Mukhlisin et al., 2021). It consists of a dual-probe detector and a small interface module that outputs an analog signal proportional to soil moisture. This design enables direct connection to the microcontroller and provides sufficiently stable readings for automated irrigation control.

• Temperature sensor (DS18B20)

Ambient temperature is monitored using the DS18B20 digital sensor, selected for its accuracy, single-wire communication, and straightforward integration with microcontroller platforms. It provides stable real-time measurements without requiring additional calibration, making it suitable for continuous environmental monitoring in the garden (Maxim Integrated, 2015).

• Water level sensor (ST045)

The ST045 analog water level sensor is used to monitor the reservoir and prevent pump operation under low-water conditions. It provides an analog output proportional to the water height, typically reaching values near 700 at maximum level and around 400 at minimum level. These readings allow the microcontroller to identify low-water states and disable the pump to avoid dry-run damage. The sensor is easy to interface, stable in continuous operation, and suitable for small-scale irrigation systems (DFRobot, 2020).

• Water pump (RS-701)

The RS-701 water pump supplies the irrigation line and provides sufficient pressure for the sprinkler system, reaching a delivery height of approximately 50 cm in the prototype configuration. This performance is adequate for small-scale garden use, although a higher-capacity model would be required for larger installations. The pump integrates easily with the microcontroller through a relay interface and operates reliably under continuous low-pressure conditions (RP Manufacturing, 2019).

• Wi-Fi module (ESP8266)

The ESP8266 Wi-Fi module is used to connect the Arduino board to the internet and enable real-time data transmission. It is compact, energy-efficient, and integrates easily with the Arduino platform due to its built-in antenna, flash memory, and support for standard network protocols. Its widespread use and large developer community further simplify debugging and firmware development, making it well suited for smart garden applications (Espressif Systems, 2018). Although the ESP8266 is capable of both data acquisition and wireless communication, a dual-board architecture was adopted in this study. The Arduino Uno handles real-time sensor reading and irrigation control, ensuring stable interfacing with multiple sensors. The ESP8266 module is dedicated to wireless communication with the server, enabling data transmission to be managed independently from time-critical sensing and actuation processes. This design improves system modularity, robustness, and maintainability while preserving the low-cost characteristic of the proposed solution.

• Fan and heating bulb

A DC fan and a heating bulb are used to regulate the garden microclimate. The fan is activated under high-temperature conditions to enhance air circulation and prevent heat stress, while the heating bulb operates at low temperatures when soil moisture is adequate. Together, these actuators provide automatic thermal regulation based on real-time sensor data, ensuring stable and favorable conditions for plant growth.

### 3.4 Circuit diagram

**Figure 8:**
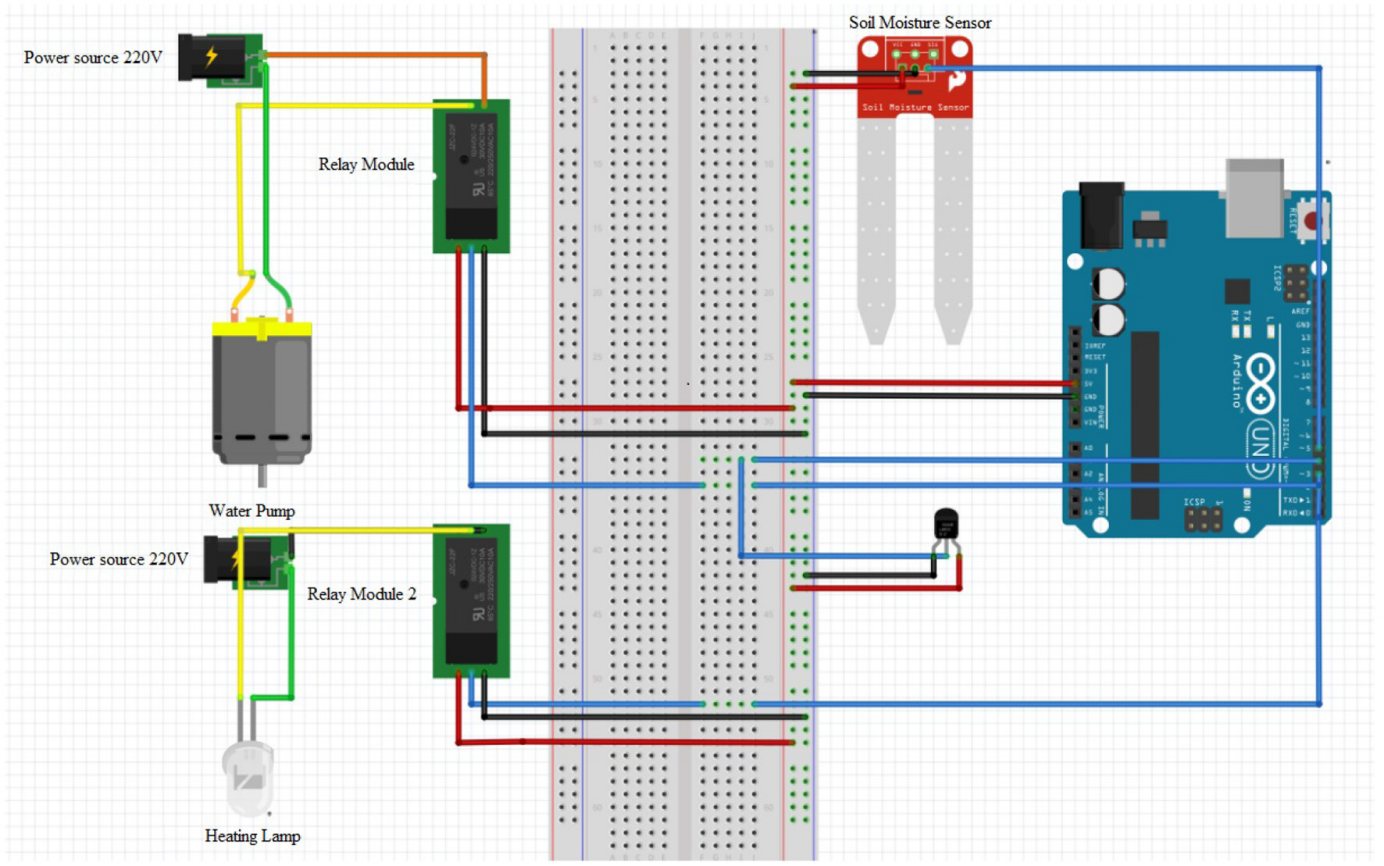
Circuit schematic of the proposed smart garden system showing connections between sensors, microcontroller, and actuators, designed using Fritzing.

Fritzing is an intuitive and widely used open-source software tool for designing and documenting electronic circuits (Knörig et al., 2009). It provides a clear and accessible interface, making the process of visualizing and building circuits both efficient and straightforward. In this project, Fritzing was employed to design the prototype circuit illustrated in Figure 8. The circuit consists of multiple sensors integrated with an Arduino board, which serves as the central control unit. The Arduino is mounted on a breadboard to facilitate connections, while the sensors are interfaced based on standard pin configurations. This schematic representation helps streamline the implementation process and ensures that the physical assembly accurately reflects the planned circuit layout.

### 3.5 Circuit integrating

**Figure 9:**
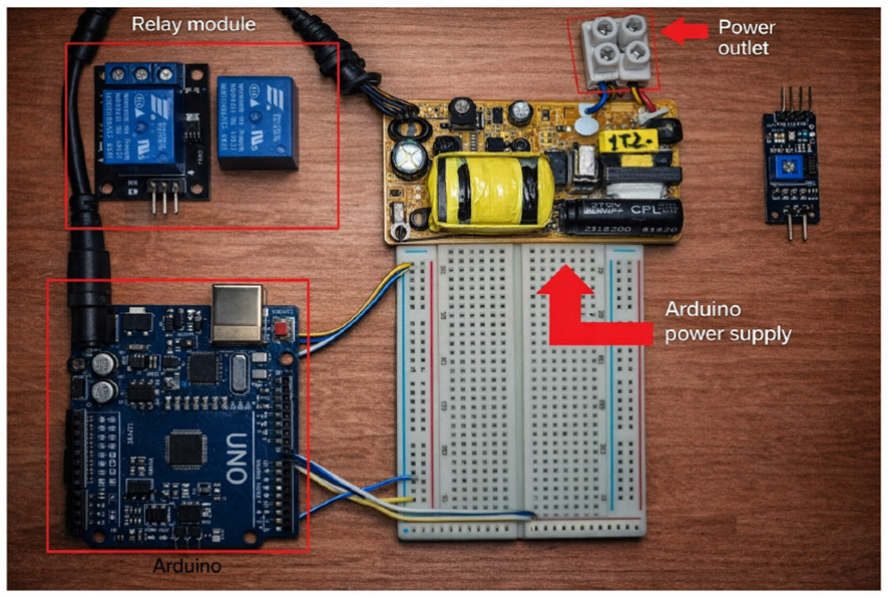
Step-by-step circuit assembly process.

**Figure 10:**
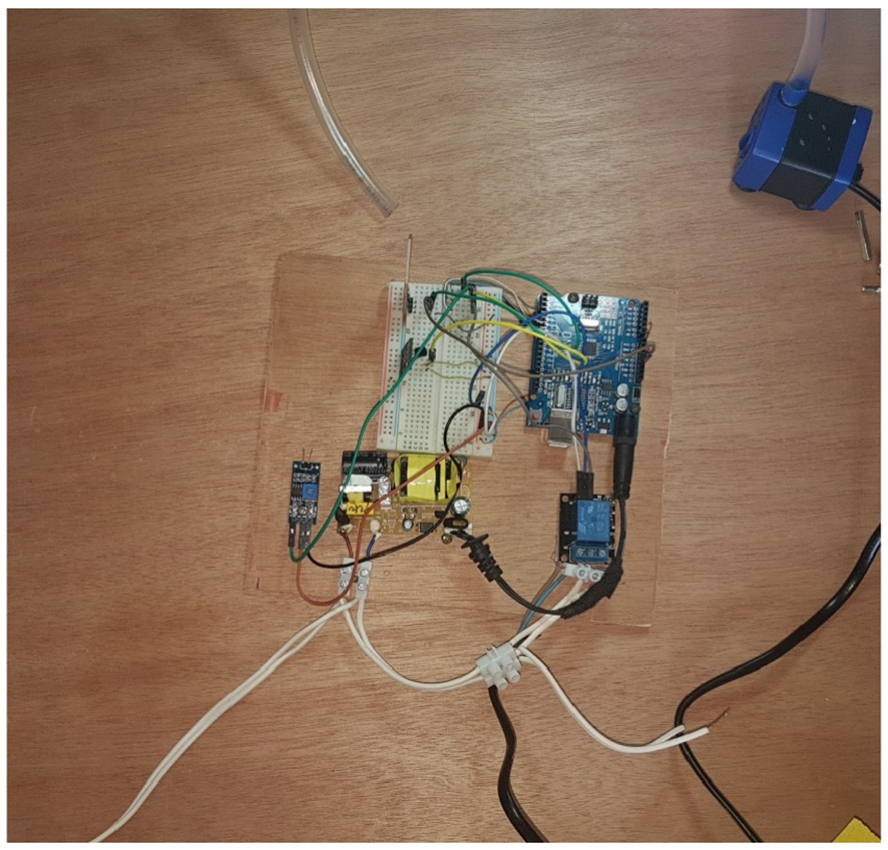
Final setup of the smart garden system showing the assembled components, including sensors, microcontroller, and actuators.

**Figure 11:**
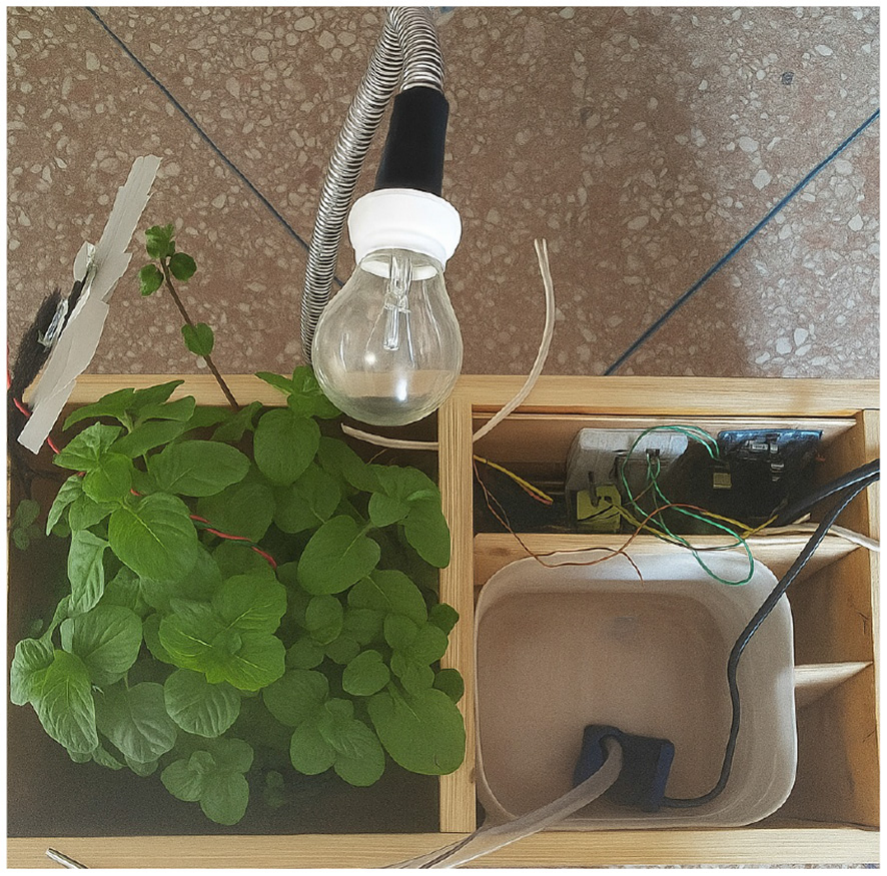
Top-down view of the smart garden system showing the plant compartment and the layout of electronic components.

The circuit was assembled step by step, as shown in Figure 9. The boards contain the essential components of the project. The system is powered by a 220V input supply. A connected transformer provides power to the Arduino, while the relays are directly powered through the Arduino's supply. Once all components were connected, the resulting setup is shown in Figure 10. The system was installed in a wooden box that houses all components, from the front panel to the sensors and indicator LEDs. The box consists of two compartments, each measuring 20 × 20 × 15 cm. The right side is designated for the plant pot, which includes the sensors and actuators (see Figure 11), while the left side contains the circuit board and water tank (also shown in Figure 11).

The circuit board is mounted on the wall of the designated compartment, allowing for easy future modifications without cluttering the space. The water tank is installed within the same section but isolated from the electronics to prevent water leakage that could disrupt the system. A top-down view of the setup, including the circuit placement, is provided in Figure 11. The entire electronics compartment is covered to ensure circuit isolation. The cover is split into two sections, enabling users to access the tank and circuit independently. With the assembly complete, the Smart Garden is now fully operational.

## 4 Results and discussion

### 4.1 Decision system results

The proposed automatic watering system was deployed for a home garden and evaluated under real operating conditions. Managed by an Arduino Uno and a set of environmental sensors, including ambient temperature and soil moisture sensors, the system processes environmental data through a fuzzy logic controller to generate autonomous irrigation decisions. Tests were conducted on two plant species, *Ocimum basilicum* (basil) and *Solanum lycopersicum* (tomato). This subsection reports the results for basil, while comparative outcomes are presented later. The fuzzy logic algorithm classifies sensors inputs into linguistic variables to support context aware decision making. For ambient temperature, the DS18B20 sensor (–55 °C to +125 °C) is mapped into three categories (Low, Medium, and High) based on commonly accepted thermal thresholds for domestic plant care (Table 1). These thresholds ensure that heating, cooling, and irrigation actions are triggered only when environmentally justified.

**Table 1:** Class of temperature.

Temperature	Class
>28 °C	High
18–28 °C	Medium
< 18 °C	Low

Soil moisture regulation follows agronomic benchmarks, particularly the field capacity (FC) and permanent wilting point (PWP). In garden soil, FC typically occurs between 25 and 35%, while PWP is reached around 10–15%, defining the available water range (AWR) (Foth, 1990). These values are translated into the fuzzy labels as shown in Table 2, enabling the system to trigger watering only when needed.

**Table 2:** Class of soil moisture.

Soil moisture	Class
>25%	High
15–25%	Medium
< 15%	Low

The ST045 sensor provides proportional readings of the tank status. A value near 700 indicates a full reservoir, whereas readings close to 450 correspond to an empty tank (Table 3). These classified inputs are processed by the fuzzy inference system to produce adaptive irrigation decisions.

**Table 3:** Class of water level.

Water level	Class
>700	Full
< 450	Empty

To evaluate the proposed system, multiple environmental scenarios were considered, including high temperature with normal soil moisture, low temperature with wet soil, and moderate temperature with dry soil. The system was tested over several months under realistic climatic variations, during which hundreds of sensor measurements were collected. Table 4 presents a representative subset of the recorded data, illustrating daily variations in ambient temperature, soil moisture, and water level. These measurements serve as inputs to the fuzzy logic controller and define the universe of discourse used in MATLAB simulations, enabling accurate modeling of a wide range of real world operating conditions.

**Table 4:** Example of data collected from sensors.

Case	Temperature (°C)	Soil moisture %)	Water level (ADC)
1	32	22	705
2	13	40	699
3	30	20	497
4	24	35	757
5	33	14	234
6	14	38	656
7	28	47	512
8	10	24	786
9	16	12	312
10	24	10	687

After defining the membership functions, fuzzy rules are formulated using simple “*IF–THEN*” statements in the MATLAB Rule Editor. These rules encode expert knowledge and govern the decision making process of the automatic watering system. As sensor data are continuously acquired, the controller evaluates whether intervention is required (namely irrigation, heating, or ventilation) through a Boolean condition referred to as garden needs care. As presented in Table 5, if no care is required, the system remains in monitoring mode. Otherwise, it proceeds to the next stage, where the appropriate actuators are activated according to the fuzzy decision. Once optimal conditions are restored, the system returns to continuous monitoring, ensuring adaptive and automatic garden management.

**Table 5:** Rules for the watering (depending on care requierements).

Condition	Output	System action
Garden needs care = no	0	Continue monitoring (no actuator engaged)
Garden needs care = yes	1	Go to next step (activate selected actuator)

In cases where the water tank is empty, as indicated by the results in Table 4, particularly scenarios 5 and 9, the system triggers an alarm and continues monitoring until the tank is refilled. Once the reservoir is full, the rule set defined in Table 6 becomes active. The fuzzy logic rules are defined using three input variables: ambient temperature, soil moisture, and water level. As shown in Table 6, the system determines the appropriate action by analyzing the various combinations.

**Table 6:** Rules for the watering system based on soil moisture and ambient temperature levels.

Soil moisture	Low temp	Medium temp	High temp
Low	Low	Medium	Low
Medium	Medium	No care required	Medium
High	Low	High	High

When the result indicates a low condition, the system activates the water pump to irrigate the soil. If the result is medium, it triggers the heating bulb to raise the ambient temperature. In cases where the result is high, indicating excessive heat and adequate soil moisture, a ventilation fan is activated to cool the environment. This adaptive response ensures optimal growing conditions for the plants at all times. Table 7 is based on results obtained through fuzzy logic processing, with Tables 1–3. serving as calibration references for sensor input. The final garden maintenance decisions are derived from fuzzy logic processing based on sensor data inputs.

**Table 7:** Solution and corresponding actuator output for each fuzzy decision result.

Fuzzy result	System action/solution
Low	Water pump (irrigation)
Medium	Heating bulb (temperature correction)
High	Fan (Cooling/ventilation)

These decisions and the corresponding results are shown in Table 8, which also reports the level of agreement of the fuzzy logic outputs with the predefined decision rules. The overall decision agreement of the system is close to 100%. The system's performance was evaluated using Equation 10, where *n* is the total number of experiments and μ(*a*_*i*_) represents the agreement degree of each individual test. Based on the experiments, the system achieved an average decision agreement of 99.2%.

**Table 8:** Fuzzy decision results and system precision for each test case.

Case	Temperature (°C)	Soil moisture %)	Fuzzy decision	Decision agreement
1	High	Medium	High	100%
2	Low	High	Medium	100%
3	High	Medium	High	100%
4	Medium	High	Medium	100%
5	High	Low	Low	100%
6	Low	High	Medium	100%
7	Medium	High	Medium	97%
8	Low	Medium	Medium	100%
9	Low	Low	Low	95%
10	Medium	Low	Low	100%


$$\begin{array}{l} \text{Accuracy}=\frac{1}{n}\overset{n}{\underset{i=1}{\sum }}\mu \left(a_{i}\right) \end{array}$$ (10)

Although the decision system results demonstrate the reliability and rule-based consistency of the fuzzy logic controller under controlled conditions, its performance must also be validated in real-world settings. Therefore, the system was deployed in outdoor garden plots with two representative plant species (basil and tomato) to evaluate its ability to maintain optimal soil moisture, reduce water usage, and preserve plant health under varying environmental conditions.

### 4.2 Experimental evaluation with basil and tomato plants

The smart garden watering system was deployed outdoors for a 30-day period, from early May to early June, to evaluate its performance under real weather conditions. Two plant species with distinct irrigation requirements were selected: *basil*, characterized by moderate water needs, and *tomato*, which requires higher water input. Each species was cultivated in two plots containing five plants each: one irrigated using a conventional threshold-based method set at 40% volumetric water content (VWC), and the other managed by the proposed fuzzy logic controller. Soil moisture and ambient temperature were continuously monitored at 5-min intervals using YL-69 and DS18B20 sensors, respectively, while rainfall data were retrieved from a nearby meteorological station for performance analysis and system evaluation during precipitation events; however, real-time irrigation decisions relied exclusively on locally measured sensor data.

**Figure 12:**
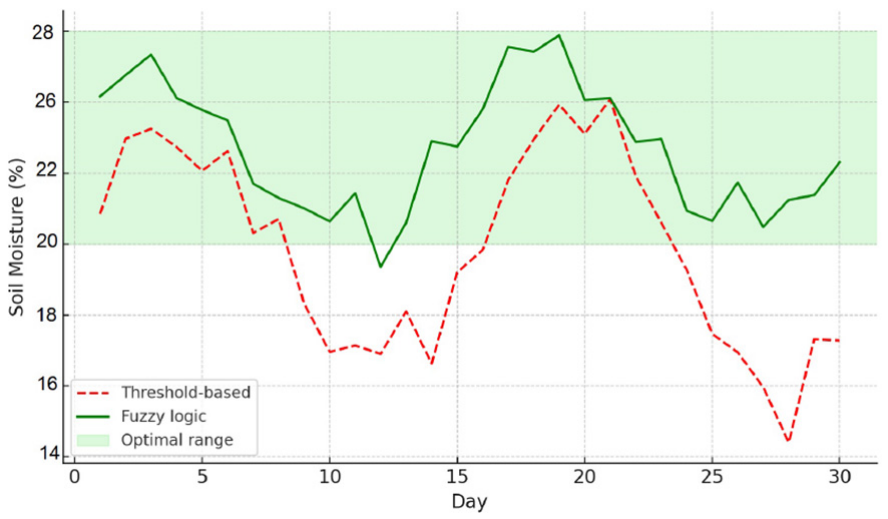
Soil moisture variation of basil plants under threshold-based and fuzzy logic irrigation control, including the optimal moisture range, during the 30-day experimental period.

**Figure 13:**
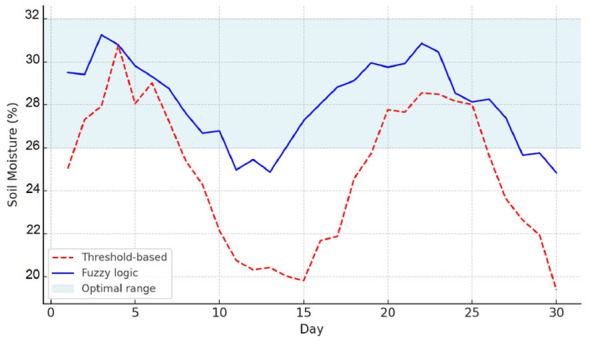
Soil moisture variation of tomato plants under threshold-based and fuzzy logic irrigation control, including the optimal moisture range, during the 30-day experimental period.

As shown in Figures 12, 13, the fuzzy logic controller maintained soil moisture within the optimal ranges for basil (20%–28% VWC) and tomato (26%–32% VWC). The results indicate that irrigation is activated when soil moisture falls below the desired range, effectively correcting moisture deficit conditions. The system maintains soil moisture within the target range with smooth and controlled variations, demonstrating stable regulation over time. Compared to the threshold-based approach, which exhibits abrupt fluctuations due to fixed control thresholds, the proposed fuzzy controller provides more gradual adjustments and improved moisture stability. Furthermore, the fuzzy system prevents prolonged moisture deficits in tomato plants during hot periods, thereby avoiding visible wilting symptoms and improving overall plant stability.

A quantitative evaluation confirms the efficiency of the proposed system. As summarized in Table 9, cumulative water consumption was reduced by 28.5% for basil and 23.8% for tomato compared with threshold-based control, without negatively affecting plant growth. Irrigation accuracy–defined as the proportion of watering events occurring within ±5% of the optimal soil moisture range–was also improved, reaching 94.2% for basil and 90.6% for tomato, compared with 81.5% and 78.3%, respectively. Paired-sample *t*-tests confirmed that both water savings (*p* < 0.01) and accuracy improvements (*p* < 0.05) were statistically significant. The system operated reliably throughout the experimental period, with no hardware or software failures observed. Minor sensor drift (±3% VWC) was detected in saline soils, indicating the need for periodic recalibration in long-term deployments. Overall, these results demonstrate that the proposed fuzzy logic controller provides a cost-effective and sustainable solution for Smart watering in home gardening (Montgomery and Runger, 2018; Walpole et al., 2012).

**Table 9:** Cumulative water usage and efficiency metrics over the 30-day period.

Plant type	Control method	Water used (L)	Water saving (%)	Irrigation accuracy (%)
Basil	Threshold-based	16.1	–	81.5
	fuzzy logic (proposed)	11.5	28.5	94.2
Tomato	Threshold-based	22.7	–	78.3
	fuzzy logic (proposed)	17.3	23.8	90.6

### 4.3 Mobile application development

**Figure 14:**
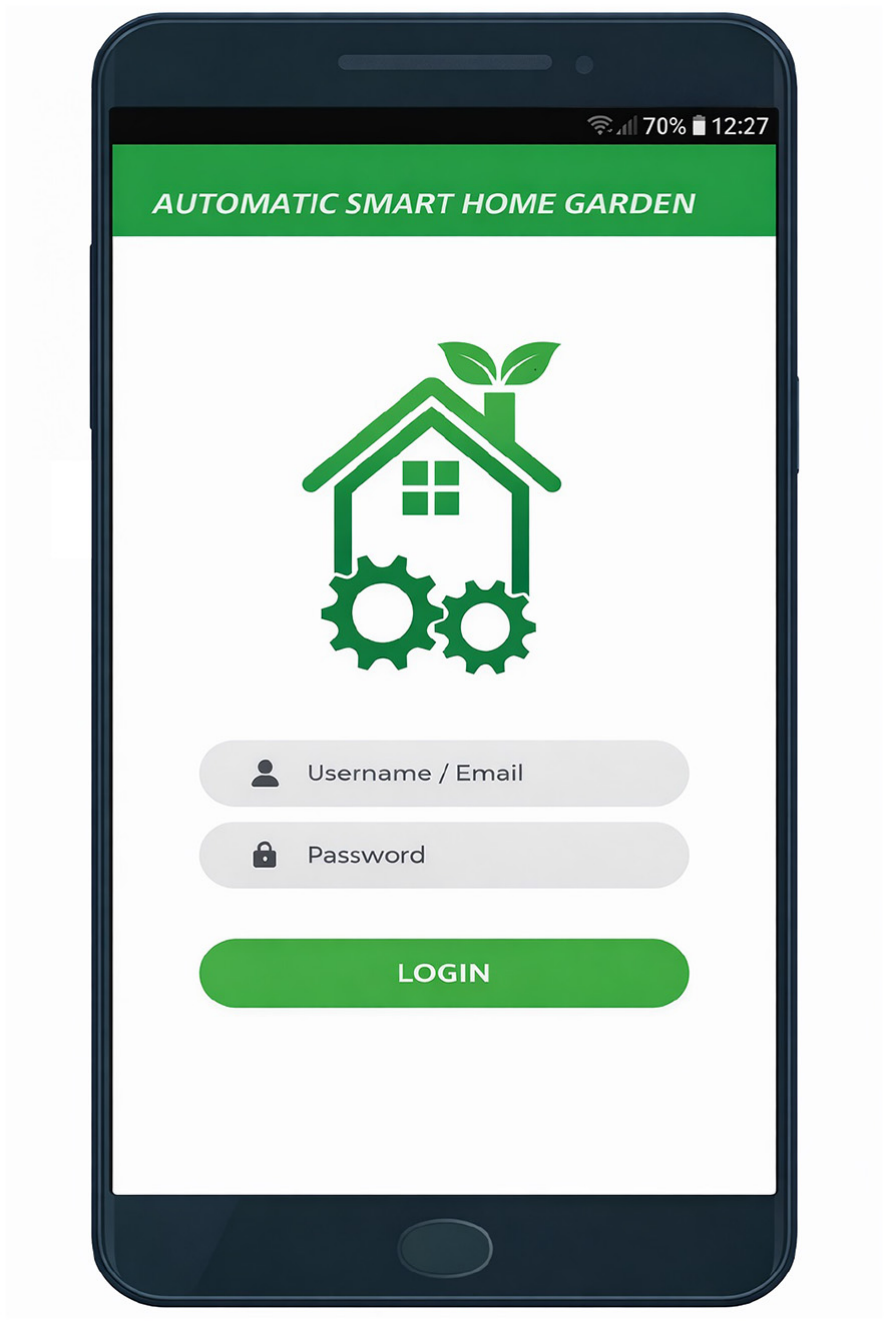
Login interface of the developed mobile application for the smart home garden system, illustrating user authentication through username/email and password fields.

**Figure 15:**
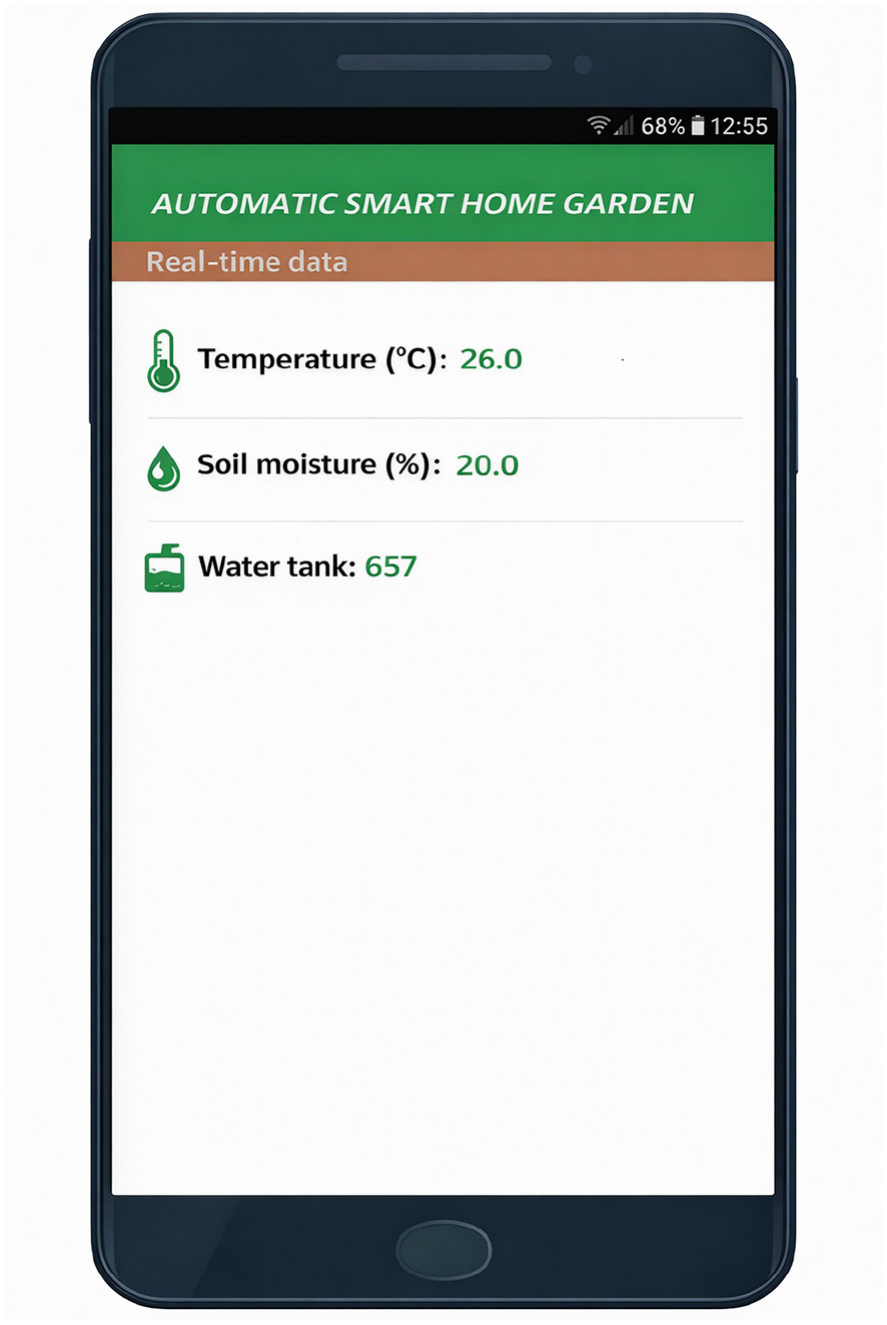
Mobile application interface illustrating real-time monitoring and visualization of key environmental parameters, including ambient temperature, soil moisture, and water level, enabling continuous observation of system conditions.

**Figure 16:**
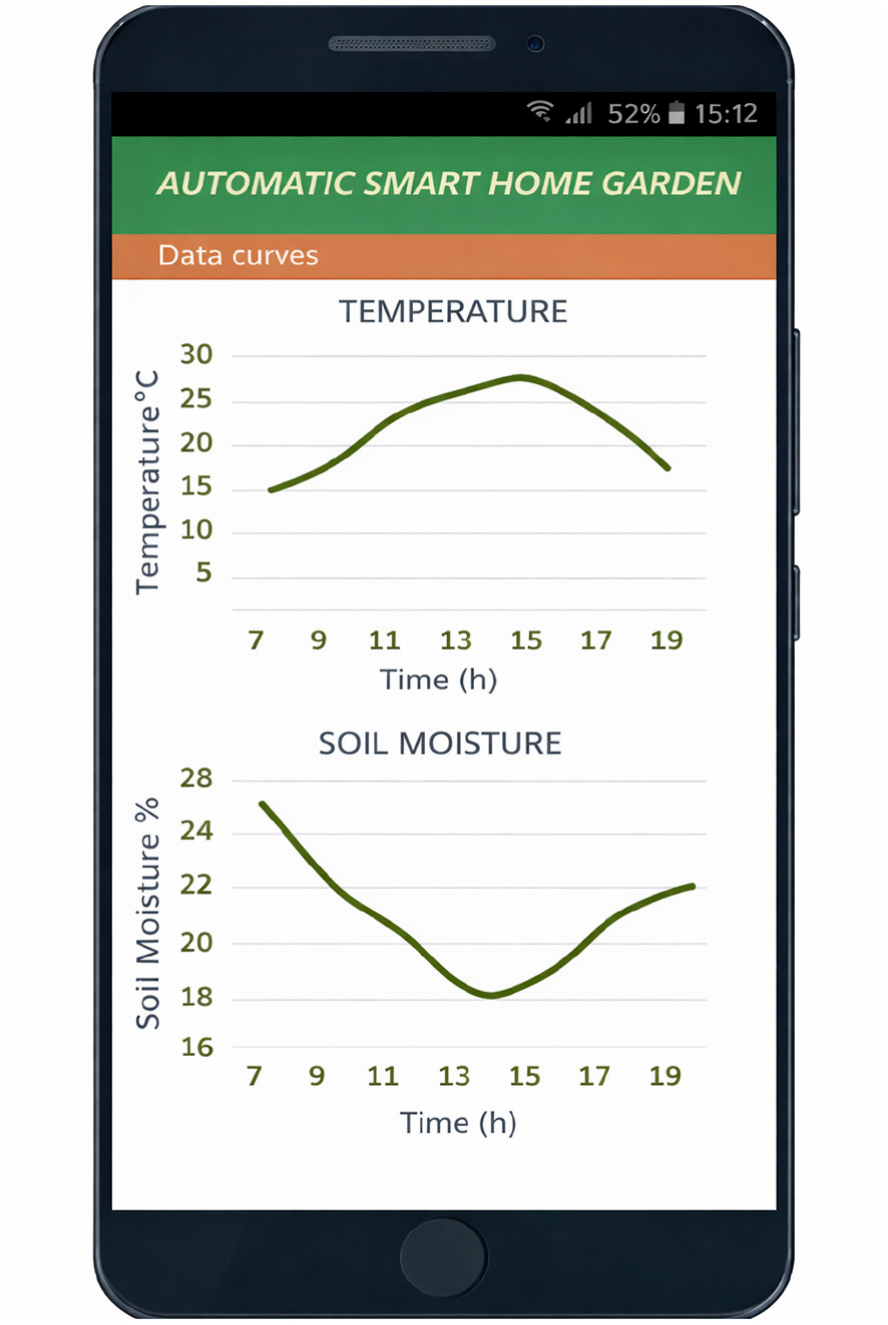
Ambient temperature and soil moisture trends recorded between 7:00 AM and 7:00 PM under fuzzy control. Temperature peaks in the mid-afternoon (around 3:00 PM), while soil moisture remains stable within optimal ranges, indicating effective regulation.

**Figure 17:**
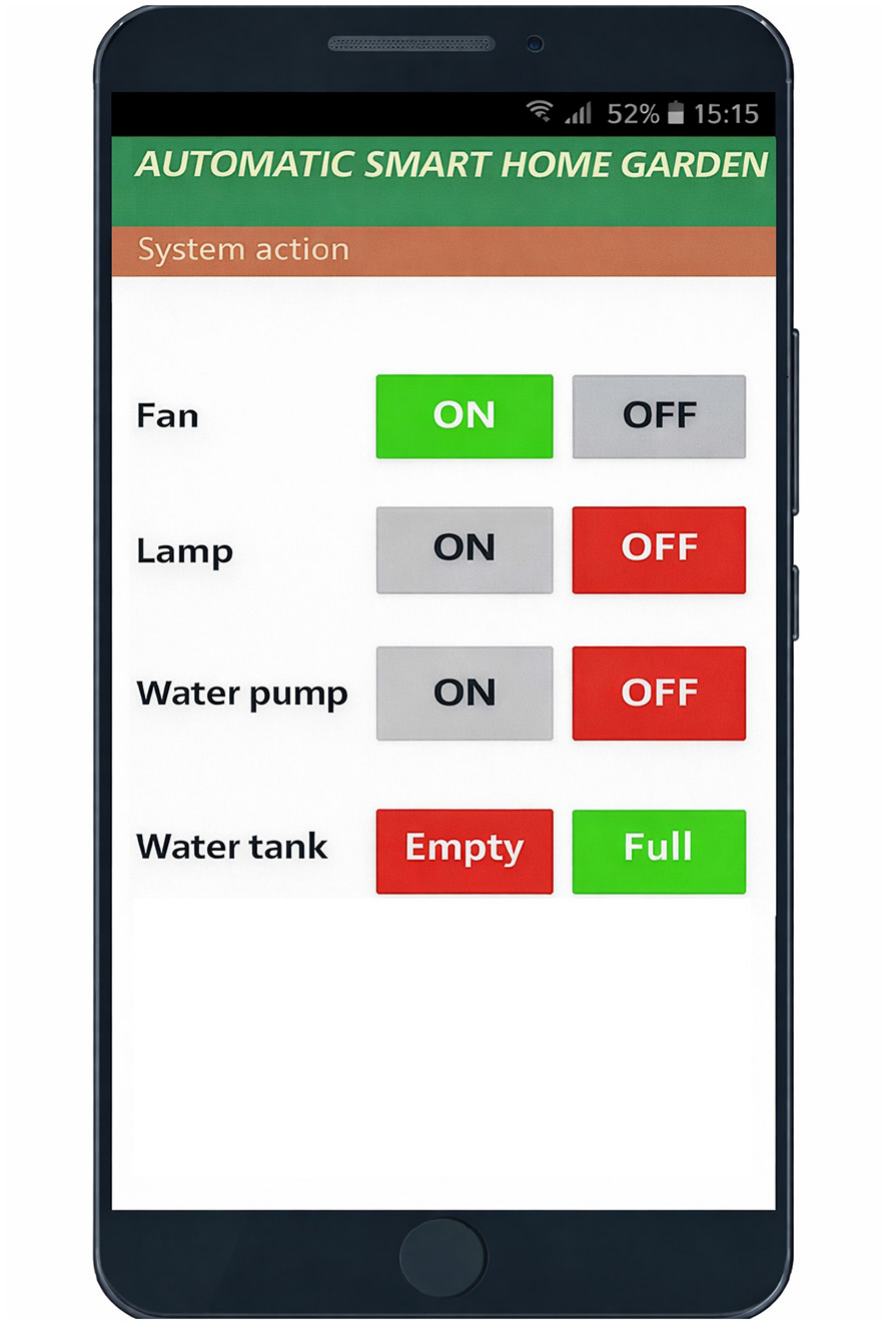
Actuator status in the mobile application, showing the fan activated, the lamp and water pump off, and a full water tank.

The proposed system includes a dedicated mobile application for remote monitoring and control of the home garden (Sanchez Rodriguez et al., 2022). The application was developed using Android Studio (2025), while the embedded firmware was implemented using the Arduino IDE (Arduino, 2025b), ensuring seamless integration between the mobile interface and the hardware platform. After secure authentication, users can access real-time sensor data and system status through a simple and user-friendly interface (Figure 14). Communication between the smartphone and the irrigation system is established over Wi-Fi using standard HTTP protocols (Fielding and Reschke, 2014), with JavaScript Object Notation (JSON) adopted as the data exchange format due to its lightweight structure and suitability for IoT applications (ECMA International, 2017). Through the mobile interface, users can monitor key environmental parameters, including ambient temperature, soil moisture, and water tank level, as well as visualize daily trends. When low soil moisture is detected, the fuzzy logic controller automatically activates the water pump and deactivates it once optimal conditions are restored. Ambient temperature regulation is achieved through dynamic activation of the fan or heating bulb in response to environmental fluctuations (Figures 15–17). The results presented correspond to the experimental deployment on (basil).

Unlike systems limited to local storage, the proposed architecture integrates ThingSpeak cloud platform using the MQTT protocol, for continuous data transmission, secure storage, and historical analysis. Sensor measurements and actuator events are synchronized between the mobile application and the cloud, enabling real-time visualization, remote notifications, and multi-device access, even in the event of local server failure. While the proposed mobile and cloud-based architecture offers significant flexibility, it remains dependent on network availability and may experience increased latency or reduced functionality under unstable Wi-Fi conditions, highlighting the need for future work on offline operation and enhanced communication robustness.

### 4.4 Comparison with existing smart watering systems

This section compares the proposed smart watering system with representative solutions from the literature, focusing on control strategy, functionality, sensing capabilities, and operational efficiency. The objective is to position the proposed approach within the current smart agriculture landscape while highlighting its key innovations. Recent smart irrigation systems have become more advanced and adaptive, combining multiple sensors, cloud platforms, and intelligent control methods. While earlier systems relied on simple threshold rules and basic irrigation control, newer approaches offer more flexible decision-making and can adjust irrigation based on real-time conditions. For instance, modern IoT-based systems use data such as soil moisture, temperature, humidity, and weather information to optimize watering and improve water efficiency (Choudhari et al., 2023; Tumpa et al., 2023). In comparison, the proposed system uses a fuzzy logic control approach combined with multiple actuators, including a water pump, a heating bulb, and a fan. This setup enables not only irrigation but also basic control of the garden environment. Unlike traditional threshold-based systems, the fuzzy controller adapts decisions smoothly based on changing conditions, reducing abrupt actions and improving overall stability. Recent studies have explored fuzzy logic and hybrid approaches for irrigation control. For example, Ambarwari et al. (2024) developed a fuzzy logic–based greenhouse system that improves water efficiency, while Arginanta and Ashari (2024) proposed an automatic watering system to enhance decision accuracy. However, these solutions primarily focus on irrigation control and typically do not include additional actuators for environmental regulation. In terms of sensing, many recent systems integrate multiple sensors such as soil moisture, temperature, humidity, and sometimes light or rainfall data (Tumpa et al., 2023; Rashid et al., 2025). In contrast, the proposed system focuses on three key parameters (soil moisture, temperature, and water level), offering a good balance between simplicity and effective decision-making. This choice keeps the system low-cost, easy to deploy, and reliable for home garden use. Another key difference is in data management and user interaction. While earlier systems relied on local storage or simple methods like SMS alerts, recent solutions use cloud platforms and mobile apps for real-time monitoring and control. In line with this, the proposed system combines a cloud platform (ThingSpeak) with an Android application, enabling real-time visualization, remote access, and historical data analysis, which improves usability and scalability. From a performance perspective, the proposed system demonstrates significant improvements in water-use efficiency and irrigation accuracy. Experimental results indicate water savings of up to 28.5% for basil and 23.8% for tomato compared with conventional threshold-based control, while maintaining optimal soil moisture levels. These results are consistent with recent findings in the literature, where intelligent control strategies have been shown to reduce water consumption and improve irrigation precision (Ambarwari et al., 2024; Tumpa et al., 2023). For comparison purposes, Table 10 summarizes the main features of the proposed system and selected representative approaches, including threshold-based, fuzzy logic–based, and IoT-enabled irrigation systems. The comparison highlights that the proposed solution combines several key advantages, including adaptive fuzzy control, multi-actuator capability, cloud integration, and mobile-based monitoring, making it a practical and cost-effective solution for smart home gardening.

**Table 10:** Comparison between the proposed system and recent smart irrigation approaches.

Feature/ metric	Proposed system	(Choudhari et al., 2023) IoT smart gardening	(Tumpa et al., 2023) IoT + AI irrigation	(Ambarwari et al., 2024) Fuzzy greenhouse	(Nabaei et al., 2025) Multimodal AI system
Control method	Fuzzy logic (adaptive)	Threshold-based	AI-based (data-driven)	Fuzzy logic (Mamdani)	Multimodal AI/data fusion
Sensors used	Soil moisture (YL-69), ambient temperature (DS18B20), water level (ST045)	Soil moisture, temperature, humidity, light	Soil moisture, temperature, weather data	Soil moisture, temperature	Multimodal (vision + environmental sensors)
Actuators	Water pump, heating bulb, fan	Water pump	Water pump	Water pump	Water pump
Data logging/processing	Continuous logging (cloud + local)	Limited	Cloud-based analytics	Local processing	Advanced data fusion
Cloud/mobile app	Yes (cloud storage + Android app)	Partial support	Yes (cloud + mobile)	No	Yes (platform-dependent)
Performance	Up to 28.5% water saving (basil), 23.8% (tomato); adaptive and stable control	Moderate improvement; basic automation	High efficiency via AI optimization	20%–25% water saving (greenhouse)	High efficiency through adaptive AI control

Despite these advantages, the proposed system still has some limitations. It relies on a limited number of inputs, such as soil moisture, ambient temperature, and water level, which may not fully capture all real garden conditions. Other factors, including sunlight, ambient humidity, and rainfall, could also influence irrigation needs. In future work, the system could be improved by integrating additional sensors and incorporating weather data to support more informed decision-making. It would also be valuable to explore more adaptive approaches, such as learning-based methods, to enhance system flexibility. Furthermore, testing the system in larger and more diverse environments will be an important next step. Other potential improvements include reducing energy consumption and strengthening data security.

## 5 Conclusion

This article presented the design and implementation of an intelligent automated watering system for home gardens, integrating an Arduino microcontroller, fuzzy logic, cloud connectivity, and a dedicated mobile application. The system autonomously manages irrigation and basic microclimate conditions using low-cost sensors to monitor soil moisture, ambient temperature, and water levels. These measurements are processed by a fuzzy logic controller that interprets environmental conditions in linguistic terms and selects the most appropriate action, such as activating the water pump for irrigation, switching on the heating bulb under cooler conditions, or operating the fan when ambient temperatures increase. A key feature of the system is the Android mobile application, supported by cloud integration, which enables real-time monitoring, alerts, secure data storage, and optional manual control. This design improves user convenience while allowing the system to operate largely in a self-sufficient manner. Compared with traditional threshold-based approaches, the proposed solution demonstrates improved adaptability and efficiency. By combining irrigation, heating, and cooling strategies, it responds more effectively to dynamic environmental conditions and reduces the risks of both over and under-watering. Experimental results indicate water savings of up to 28.5% compared with conventional threshold-based systems. Overall, the study demonstrates that smart garden technologies can be both effective and affordable for everyday users. Future work will focus on integrating additional sensors and leveraging AI-based methods to improve decision-making, increase adaptability, and enhance the system's performance across diverse environments.

## Data Availability

The original contributions presented in the study are included in the article/supplementary material, further inquiries can be directed to the corresponding author.
